# Development of neuronal timescales in human cortical organoids and rat hippocampus dissociated cultures

**DOI:** 10.1152/jn.00135.2024

**Published:** 2024-07-17

**Authors:** Blanca Martin-Burgos, Trevor Supan McPherson, Ryan Hammonds, Richard Gao, Alysson R. Muotri, Bradley Voytek

**Affiliations:** ^1^Department of Cognitive Science, University of California, San Diego, La Jolla, California, United States; ^2^Halıcıoğlu Data Science Institute, University of California, San Diego, La Jolla, California, United States; ^3^Neurosciences Graduate Program, University of California, San Diego, La Jolla, California, United States; ^4^Kavli Institute for Brain and Mind, University of California, San Diego, La Jolla, California, United States; ^5^Department of Pediatrics/Rady Children’s Hospital San Diego, School of Medicine, University of California, San Diego, La Jolla, California, United States; ^6^Department of Cellular & Molecular Medicine, School of Medicine, University of California, San Diego, La Jolla, California, United States

**Keywords:** cortical organoids, development, local field potentials, rodent hippocampal dissociated cultures, neuronal timescales

## Abstract

To support complex cognition, neuronal circuits must integrate information across multiple temporal scales, ranging from milliseconds to decades. Neuronal timescales describe the duration over which activity within a network persists, posing a putative explanatory mechanism for how information might be integrated over multiple temporal scales. Little is known about how timescales develop in human neural circuits or other model systems, limiting insight into how the functional dynamics necessary for cognition emerge. In our work, we show that neuronal timescales develop in a nonlinear fashion in human cortical organoids, which is partially replicated in dissociated rat hippocampus cultures. We use spectral parameterization of spiking activity to extract an estimate of neuronal timescale that is unbiased by coevolving oscillations. Cortical organoid timescales begin to increase around *month 6* postdifferentiation. In rodent hippocampal dissociated cultures, we see that timescales decrease from in vitro *days 13*–*23* before stabilizing. We speculate that cortical organoid development over the duration studied here reflects an earlier stage of a generalized developmental timeline in contrast to the rodent hippocampal cultures, potentially accounting for differences in timescale developmental trajectories. The fluctuation of timescales might be an important developmental feature that reflects the changing complexity and information capacity in developing neuronal circuits.

**NEW & NOTEWORTHY** Neuronal timescales describe the persistence of activity within a network of neurons. Timescales were found to fluctuate with development in two model systems. In cortical organoids timescales increased, peaked, and then decreased throughout development; in rat hippocampal dissociated cultures timescales decreased over development. These distinct developmental models overlap to highlight a critical window in which timescales lengthen and contract, potentially indexing changes in the information capacity of neuronal systems.

## Graphical abstract

**Figure:**
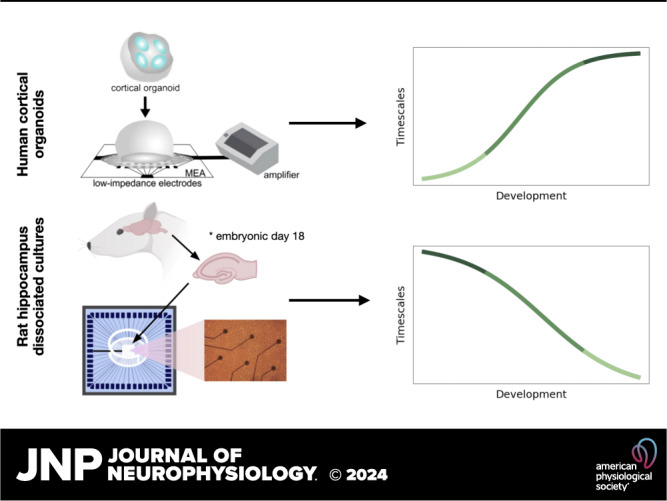

## INTRODUCTION

Every moment of our conscious lives is captured by our senses. Complex cognition, such as working memory and decision-making, requires maintaining information across timescales that span several orders of magnitude—from milliseconds to decades. How neuronal circuits are able to flexibly preserve information in their network dynamics over varying durations, even in the absence of any continuing sensory input, remains an unanswered question. The neuronal timescale—the duration over which the activity of a neuronal population typically persists—is an intrinsic feature of neuronal circuits and has the potential to explain how information might be maintained over multiple temporal scales throughout neuronal systems (1). Neuronal timescales exhibit hierarchical spatial organization, are functionally dynamic, and relate to gene expression patterns (1, 2). These features are thought to enable the integration of information across multiple temporal scales, reflecting the “information capacity” of neuronal circuits and allowing for the emergence of cognitive functions dependent on signals from the past and predictions for the future.

Despite growing interest in the potential of neuronal timescales to explain cognitive functioning (1, 3–13), how these timescales emerge and change over an organism’s development remains unexplored. Understanding how neuronal timescales develop would help elucidate how multiscale dynamics across neuronal circuits support complex cognition. However, studying long-term changes in neuronal circuit dynamics proves challenging due to the limitations of existing model systems: chronic invasive data acquisition cannot be performed on developing human brains, and although animal models are powerful for understanding neuronal circuitry, they have distinct network organization, and they exhibit different cognitive functions.

Recently, brain cortical organoids generated from human-induced pluripotent stem cells (hiPSCs) have emerged as a promising three-dimensional model for the study of the human cortex (14–20). This model lends itself to the study of development because cortical organoids are generated from pluripotent stem cells by administering external growth factors that emulate the neural developmental environment observed in utero. Brain organoids recapitulate epigenetic and transcriptional features displayed in human fetal brains across development (16, 21). In addition, cortical organoids display rhythmic patterns of electrical activity resembling the electrical activity in the brain of preterm infants (22), illustrating their ability to model the development of cortical dynamics.

**Figure 1.**
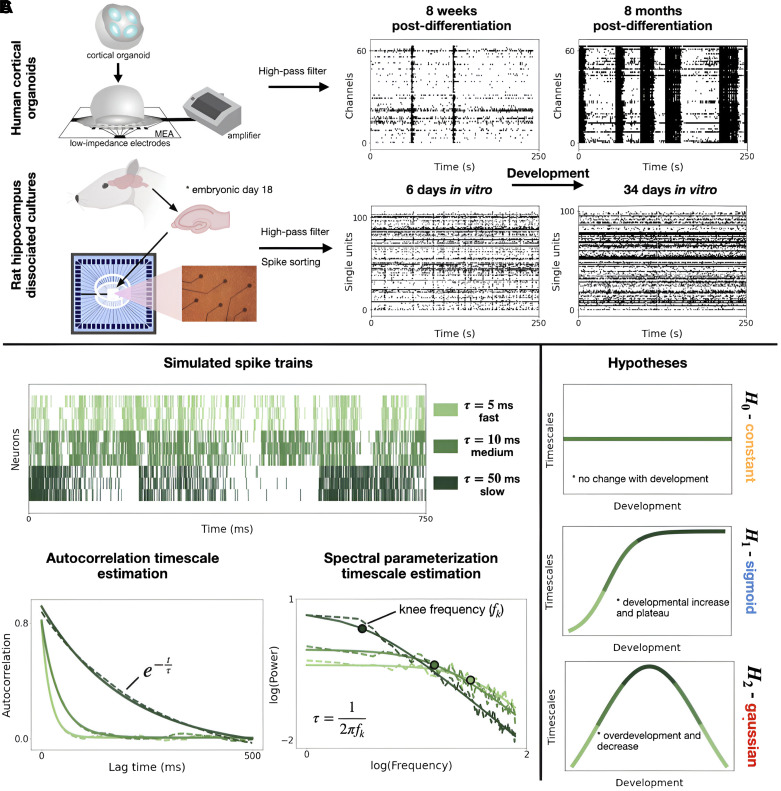
Timescale estimation over development in cortical organoids and rat dissociated hippocampal cultures. *A, left*: experimental setup for cortical organoid multielectrode array (MEA) recordings and single-unit recordings of rat dissociated cultures from extracted hippocampi of *day 18* embryos. *Right*: example spike raster plots earlier and later in development for each dataset. *B*: timescale estimation methods on simulated spiking data with different ground-truth timescales (5 ms, 10 ms, and 50 ms): in the autocorrelation method, the neuronal timescale is the decay time constant (τ) of an exponential function fit (solid line) to the autocorrelation function (ACF, dashed line). Alternatively, in the spectral parameterization method, neuronal timescales can be estimated from the knee frequency of the aperiodic component of a PSD. Timescales estimations using the autocorrelation method are biased by the presence of oscillations in the data, whereas estimations from the spectral parameterization methods are not (Supplemental Fig. S1). *C*: hypotheses for the developmental trajectory of timescales. H0 (constant model): timescales do not change over development. H1 (sigmoid model): timescales initially increase and then plateau over development. H2 (Gaussian model): timescales increase and decrease over development.

**Figure 2.**
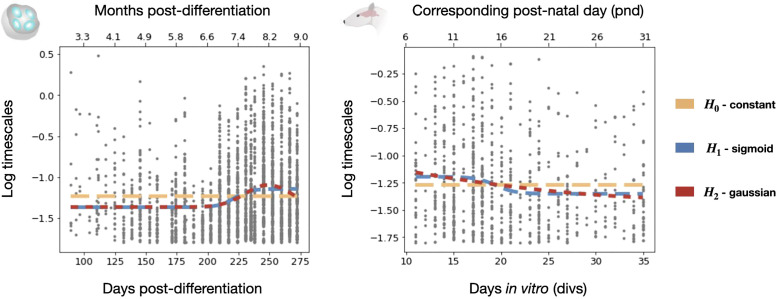
Timescales fluctuate nonlinearly over development. To test our hypotheses for the developmental trajectory of timescales, we fit constant offset, sigmoid, and Gaussian functions to spectral parameterization timescale estimation results. Cortical organoids (*left*) show an increase in timescales with a slight decrease toward the end of the developmental window examined. Rat dissociated hippocampal cultures (*right*) show a decrease in timescales followed by stabilization at a decreased value.

Here, we report on the developmental trajectory of intrinsic timescales in multiunit multielectrode array (MEA) spiking data from cortical organoids (Fig. 1*A*, *top*). We contextualize these findings by characterizing timescale development in an openly available data set from another neurodevelopmental model, rodent hippocampal dissociated cultures (Fig. 1*A*, *bottom*), to show the applicability of this analysis approach across diverse developmental systems. These two model systems span distinct species, brain regions, and developmental periods. Acknowledging these differences, we gain insight into a diversity of changing trajectories exhibited by neuronal timescales, and show their dependence on development in multiple contexts. We show, in both models, that timescales fluctuate nonlinearly over development (Fig. 2).

## MATERIALS AND METHODS

### Experimental Details

#### Generation of human cortical organoids and MEA data collections.

The cortical organoid data used was originally published by Trujillo et al. (22), though the analyses presented here are novel and unrelated to the findings of that publication. See Ref. 22 for a detailed description of the organoid development, validation, care protocol, and multielectrode array data recordings. After 6 wk of development, cortical organoids were plated in 12 wells in an MEA recording plate (Axion Biosystems, Atlanta, GA). Each well contained 64 low-impedance (0.04 MU) platinum microelectrodes with 30 mm of diameter spaced by 200 mm. Eight of the 12 wells were recorded, yielding a total of 512 data channels over recording. Recordings were collected on a weekly basis starting 2 wk after plating or 8 wk post-organoid differentiation. A Maestro MEA system and AxIS Software Spontaneous Neural Configuration (Axion Biosystems) were used for recording data at 12,500 Hz, along with a custom script for online band-pass filtering from 0.1 Hz and 5 kHz. Approximately 4 min of data were acquired during each recording session.

#### Single-unit rat dissociated culture (CRCNS HC-8 data set).

The single-unit rat hippocampal dissociated culture data set analyzed in this paper is openly available on CRCNS (hc-8 data set) and was originally published by Timme et al. (23, 24). To obtain the dissociated cultures in the data set, rat embryos were extracted at *embryonic day 18*. The hippocampi of these embryos were extracted, combined, and plated as dissociated cultures on large multielectrode arrays (Multichannel Systems, square lattice, 60 electrodes, 200-μm interelectrode spacing, 30-μm electrode diameter, flat electrodes). Recordings of spontaneous activity were performed at 20 kHz sampling rate for *days in vitro* (*DIV*) *6* through *35*, with all cultures being recorded multiple times throughout development. A total of 435 recordings of ∼1 h are provided in the data. Most recordings include an average of 100 neurons with an average rate of 1.9 Hz. Further information about the experimental details of this data set are described in Refs. 23 and 24.

### Methodological Details

All data were analyzed in Python. Statistical analyses were performed using the Scipy (25) and scikit-learn packages (26), with electrophysiological data analyses performed using the NeuroDSP (27), and specparam (28) toolboxes. Additional analyses were performed using custom Python scripts, all of which are available on the laboratory GitHub account in https://github.com/voytekresearch/timescale_development.

#### Cortical organoid data preprocessing.

Recoded data were preprocessed in Matlab. In short, data across channels in a well were referenced to the median value for a given time point, a third-order Butterworth bandpass filter from 300 to 3,000 Hz was applied, and an adaptive threshold of 5.5 deviations from a median estimated standard-deviation was used to determine spike timings (29). The onset of each spike was defined as the peak time after the absolute value of the signal crossed the threshold, with an enforced 1-ms delay between spikes. Because of the limited spatial resolution of the MEA electrodes, spikes do not represent putative single-unit activity, and multiunit activity for individual channels was used in all subsequent analyses.

Network events—time windows in which spiking activity was increased—were identified for each recording. For each well in a given recording, spikes were summed across channels into a population vector. Network events were defined around bursts of spiking activity that exceeded one-half of the maximal spiking activity in a given recording. A window around the onset of each bust was extracted, starting 0.5 s before and extending 2.5 s after. Data from these windows of activity were analyzed with respect to individual channels in all subsequent analyses. Our analyses were restricted to network events as spiking activity at all other points in the recordings was very sparse, limiting our ability to compute autocorrelations and power spectra and hence, timescales.

#### Rat hippocampal dissociated cultures data preprocessing.

Single-unit spikes were binned into bins of 100 ms. To obtain population spiking for further timescale estimation analyses, we summed all binned single-unit spiking vectors into a single population spiking vector. To estimate the postnatal developmental period modeled by the dissociated cultures, we calculated postnatal equivalence days. The average gestation period of rats is ∼23 days, and the dissociated cultures were generated from *embryonic day 18* rat embryos. Therefore, estimated postnatal equivalence days of divs (days in vitro) were calculated using the following formula:

$$\begin{array}{l} \text{postnatal day}=\text{divs }-\;(\text{gestation period }-\;\text{embryonic extraction day})\\ =\text{divs}-(23-18)\\ \text{postnatal day = divs}-5 \end{array}$$

Given these calculations, this data set models an early postnatal developmental period in rats, from *postnatal day 1* (*divs 6*) to *postnatal day 30* (*divs 35*).

#### Autocorrelation timescale estimation.

Autocorrelation functions for binned spiking data (100-ms bins) are calculated using the statsmodels python package (30) with 100-ms lags and 3,000-ms windows. Timescales are estimated from the exponential decay functions fit to the computed autocorrelations of spiking data. In this approach, timescales (τ) are the decay time constants of the exponential decay functions ($e^{-\frac{t}{\tau }})$.

#### Spectral parametrization timescale estimation.

We apply spectral parameterization, using specparam (28) to extract periodic and aperiodic components from PSDs of spiking data. Briefly, we decompose log-power spectra into a summation of narrowband periodic components—modeled as Gaussians—and an aperiodic component—modeled as a generalized Lorentzian function centered at 0 Hz. Power spectral density (PSD) is estimated using Welch’s method (31) using 1.0-s Hamming windows with 0.5-s overlap. Custom functions for this can be found in NeuroDSP (27), an open-source digital signal processing (DSP) toolbox for neural time series (neurodsp.spectral.compute_spectrum). Neuronal spiking timescales were estimated from the knee frequency (*f_k_* = *k*^1/χ^, where *k* is knee parameter and χ is power spectral exponent) of the aperiodic component via the following equation:

$$\tau =\frac{1}{2\pi f_{k}}$$

#### Cortical organoid timescale estimation.

We computed power spectra and autocorrelation functions over bursts of multiunit cortical organoid activity (network events) collected across MEA channels. Spectral and autocorrelation parameterization were used, as previously described to estimate neuronal timescale for each channel at a given time point. Extreme outliers (timescales with a knee frequency greater than 100 or less than 0.01) were removed. The remaining values for each well were log-transformed, and further outliers (defined as values that were more than 4 standard deviations from the mean at a given time point) were removed. Neuronal timescales were estimated for 64 channels in eight wells over 39 recording days spanning 8 wk, producing a timescale developmental trajectory.

#### Rat dissociated cultures timescale estimation.

We computed power spectra and autocorrelation functions over segments of recordings for individual single-unit activity collected from rodent hippocampal dissociated cultures. Spectral and autocorrelation parameterization were used as previously described to estimate neuronal timescale for each single-unit at a given time point. Timescale estimates were averaged across single-units within a given culture to produce a single value per culture. Extreme outliers (timescales values over 100 ms) were removed for all calculations. Neuronal timescales were estimated for a total of 28 cultures over 435 recordings from 6 days in vitro to 35 days in vitro, producing a timescale developmental trajectory.

#### Fitting of timescale developmental trajectories.

Constant (horizontal offset), sigmoidal, and Gaussian functions were fit to the average timescale trajectory across the eight organoid cultures to quantify developmental trends. We fit the functions to the calculated mean of the timescales at each recording time point (rat culture days in vitro or organoid days postdifferentiation). All functions were fit using Scipy’s curve fit function (25). We provided initial guesses based on the timescale trajectory when fitting the sigmoid and Gaussian functions. To compare the three models, we computed pairwise *f* tests to obtain *f* ratios and *P* values.

#### Simulation of spike trains with specific timescales.

We simulate the aperiodic background component of neural field potential recordings as autocorrelated stochastic processes by convolving Poisson population spikes with exponentially decaying synaptic kernels with predefined decay time constants using NeuroDSP (27) (neurodsp.sim.sim_synaptic_current). To simulate spiking data with added oscillations, sine oscillations were added to the spiking probabilities obtained after convolving the Poisson population spikes with the kernels. Autocorrelations and PSDs of the simulated data are computed and parameterized as described earlier.

#### Calculation of total oscillatory power.

Total oscillatory power is calculated for rat culture single units and cortical organoid electrode channels using the results obtained from spectral parameterization. For every unit and channel at every recording time point, the power of all the peaks found in the periodic component of the PSD is summed to obtain the total oscillatory power. We calculated the mean and standard deviation of the oscillatory power for every time point across all units/channels.

## RESULTS

Neuronal timescales are traditionally estimated from the autocorrelation function (ACF) of binned spiking data (1). Here, the neuronal timescale is the decay time constant (τ) of an exponential function fit to the ACF. Recent work has demonstrated that biases in neuronal timescale estimation can arise from a limited number of samples (the finite duration of signals) and the presence of oscillatory activity that can be seen in the ACF (13). Alternatively, neuronal timescales can be estimated in the frequency domain from the knee frequency of the aperiodic component of a power spectrum. The timescale (decay constant) can be computed as $\tau =\frac{1}{\;2\pi k^{1/\chi }}$ where *k* (knee parameter) and χ (power spectral exponent) combine to produce the knee frequency (2). Because this approach involves the isolation of aperiodic neuronal activity, it avoids the biases introduced by oscillatory dynamics, which are known to be nonstationary over cortical organoid development (22). We apply both methods to the analyzed datasets (Fig. 1*B*), reporting the spectral timescale estimation in the main text (Fig. 2) and discussing the autocorrelation timescale estimation and its associated bias in the Supplemental Materials.

We compared three plausible hypotheses for how timescales change with development: (H0) timescales remain constant, (H1) timescales show a sigmoidal increase-to-plateau pattern, (H2) timescales follow a Gaussian trajectory, increasing and then decreasing (Fig. 1*C*). These hypotheses were derived from the developmental trajectories of network-level electrophysiological properties previously observed in cortical organoids (21). We test these hypotheses by fitting constant offset, sigmoidal, and Gaussian functions to the developmental trajectories collected from cortical organoid and rodent hippocampal cultures (Fig. 2).

In cortical organoids, we see an overall increase in timescale with development. The Gaussian and sigmoidal functions best captured the variance in the data [Gaussian vs. constant *F* test: *F*(3,2367) = 42.6, *P* = 1.11 × 10^−16^; sigmoid vs. constant *F* test: *F*(3,2367) = 37.3, *P* = 1.11 × 10^−16^], and no difference was found in the variance explained between the sigmoidal and Gaussian functions [Gaussian vs. sigmoid *F* test: *F*(0,2367) = 1.01, *P* = 0.438]. Timescales increase throughout the first part of development, with the fitted Gaussian function peaking around *day ∼250*, indicating that timescales began to decrease over the last ∼25 days of development. We examined this change at the end of the measured developmental window, confirming that the distribution of timescales at the timepoint just before the fitted Gaussian’s peak and final timepoint are statistically distinct [independent samples *t* test: *t*(379) = 5.69, *P* = 2.61 × 10^−8^]. These results demonstrate that timescales do not increase monotonically.

To see if these results replicated, we analyzed an additional, independent data set of dissociated hippocampal cultures from rats. Here, we observed a decrease in timescale over development. The Gaussian and sigmoidal functions both captured more variance than the constant function [Gaussian vs. constant *F* test: *F*(2,762) = 9.02, *P* = 1.34 × 10^−4^; sigmoid vs. constant *F* test: *F*(3,761) = 6.84, *P* = 1.49 × 10^−4^]. There was no significant difference between the Gaussian and sigmoid functions in terms of variance explained [Gaussian vs. sigmoid *F* test: *F*(1,761) = 2.45, *P* = 0.118]. Despite the comparably good performance of the Gaussian model, timescales begin decreasing at the start of the developmental window sample, making the Gaussian hypothesis inconclusive.

## DISCUSSION

Examining two experimental model systems with clear distinctions enables us to contrast developmental features that span different species (human vs. rat) and brain regions (cortical vs. hippocampal networks). When considering the differences between the two datasets, it is important to note that these models likely capture different stages of neurodevelopment. Cortical organoids are thought to recapitulate gene expression, epigenetic profiles, cellular diversification, and population dynamics of prenatal cortical human development (14, 16–22). In comparison, the rodent hippocampal dissociated cultures are believed to describe rat postnatal development. To estimate the postnatal developmental period modeled by the dissociated cultures, we calculated postnatal equivalence days (see *Rat hippocampal dissociated cultures data preprocessing*). Since the average rat gestation time is 21–23 days (17), and the cultures were produced from *embryonic day 18* embryos, we could equate in vitro *day 6* to approximately *postnatal day 1–3*. When examining key developmental benchmarks by age across humans and rodents, developmental milestones in rodent *postnatal days 1–3* are comparable with those of human preterm infants, and milestones in rodent *postnatal days 25–35* are comparable with those of 4- to 11-yr-old humans (32). The developmental timelines of our examined models compared with human development suggest that our timescale developmental trajectories potentially share some overlap, where late organoid development is analogous to early postnatal rodent culture development. Interestingly, the decrease in organoid timescales at the end of development potentially maps onto the decrease we see in early rodent slice development.

**Figure 3.**
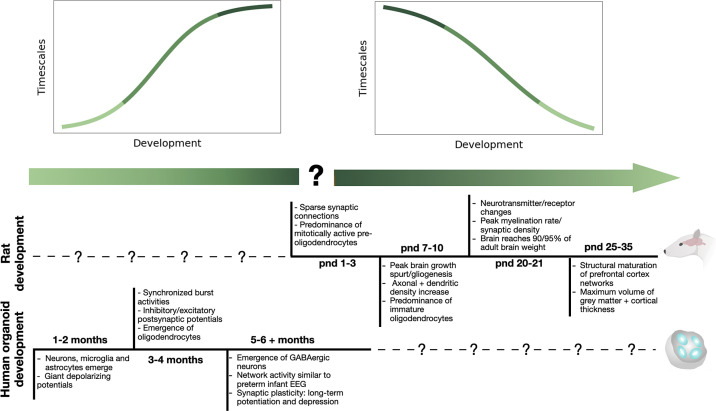
Timescale trajectory over development may coincide with key developmental milestones in rats and human organoids. *Top*: proposed timescale trajectory over prenatal and early postnatal development. *Bottom*: timeline of key developmental milestones in rat development (32) and human organoid development (33).

Our results suggest that neuronal timescales begin to increase near *month 6* of human cortical organoids, overlapping with electrophysiologic changes and the emergence of GABAergic network inhibition (22, 34, 35). The onset of the Gaussian trajectory seen in late organoid development potentially corresponds to the decrease seen in the rodent hippocampal cultures (approximately culture in vitro *days 13–23* and rodent *postnatal days 7–17*). This aligns with key rodent development milestones such as peak brain growth, peak gliogenesis, and increasing axonal and dendritic density. We postulate that together, these trajectories might follow a generalized model (Fig. 3) in which timescales begin to lengthen during mid-late prenatal development, overshoot in a Gaussian-shape after birth, and then decline and plateau to a steady state. Though more research is needed to confirm this hypothesis, a generalized developmental trajectory could support the gradual emergence and consolidation of “information capacity” within neuronal networks, allowing circuits to maintain information across time.

Though the developmental timelines of the models overlap, affording the possibility of a generalized developmental trajectory for timescales, we acknowledge that timescales are estimated from distinct types of neuronal networks in each of our model systems. The cultured cells analyzed here come from different species (human vs. rat), brain regions (cortical vs. hippocampal), and culture structures [three-dimensional (3-D) vs. two-dimensional (2-D)]. These factors all have the potential to influence development of timescales, as they all induce distinct cellular compositions, connectivity structure, and functional interactions within cultured populations. Due to the limited activity generated throughout development in 2-D cortical organoid cultures (22), 3-D cortical organoids afforded us the possibility of examining timescales throughout development, into a range of overlap with other developmental models such as rodent-dissociated cultures. Future work developing 3-D organoid models in other species targeted to reproduce the characteristics of specific brain regions is needed to further enrich our ability to contrast distinct neurodevelopmental model systems.

Recent work examining the sequential structure of population activity suggests that cortical organoids are uniquely situated to examine the brain’s intrinsic network dynamics. Sedmak et al. (36) show that 3-D cortical organoids develop a flexible repertoire of nonrandom spiking sequences, whereas 2-D dissociated cortical cultures do not. Interestingly, slices obtained from murine somatosensory cortex—as opposed to 2-D derived cultures—also demonstrated flexible yet nonrandom spiking, further supporting the notion that a 3-D structure is required to enable network dynamics in vitro. This work highlights how a network’s information capacity can develop in the absence of experience, suggesting it is an intrinsic feature of cortical networks. We likewise posit that the development of timescales in cortical organoids is an intrinsic (nonexperience-dependent) feature of neuronal populations, and that timescale lengthening suggests the presence of a critical developmental window during which cognitive capacity increases. This stands in contrast to the observation that the timescale of populations of neurons is likely not an intrinsic feature of the population, but is likely dynamic and task and/or state dependent (2). Future studies tracking the detailed population spiking structure of cortical organoids with high-density (HD) MEA systems and their perturbation via stimulation would enable a mechanistic understanding of how cortical capabilities emerge.

## DATA AVAILABILITY

All code used for all analyses and plots are publicly available on GitHub at: https://github.com/voytekresearch/timescale_development.

## SUPPLEMENTAL MATERIAL

10.6084/m9.figshare.25970236 Supplemental Materials: https://doi.org/10.6084/m9.figshare.25970236.

## GRANTS

Funding was provided by a California Institute for Regenerative Medicine (CIRM) training grant EDUC4-12804 (to B.M.-B.), Department of Defense W81XWH2110306 (to A.R.M.), and an NIH National Institute of General Medical Sciences Grant R01GM134363-01 (to B.V.).

## DISCLOSURES

A.R.M. is a cofounder and has an equity interest in TISMOO, a company dedicated to genetic analysis and brain organoid modeling focusing on therapeutic applications customized for autism spectrum disorder and other neurological disorders with genetic origins. The terms of this arrangement have been reviewed and approved by the University of California San Diego in accordance with its conflict-of-interest policies. A.R.M. is an inventor of several patents on human functional brain organogenesis, including the protocol used here. None of the other authors has any conflicts of interest, financial or otherwise, to disclose.

## AUTHOR CONTRIBUTIONS

R.G., A.R.M., and B.V. conceived and designed research; B.M.-B., T.S.M., R.H., and B.V. analyzed data; B.M.-B., T.S.M., R.H., and B.V. interpreted results of experiments; B.M.-B. and T.S.M. prepared figures; B.M.-B. and T.S.M. drafted manuscript; B.M.-B., T.S.M., R.H., R.G., A.R.M., and B.V. edited and revised manuscript; B.M.-B., T.S.M., R.H., R.G., A.R.M., and B.V. approved final version of manuscript.
